# Emergency Physician Perspectives on the Use of Patient Experience Surveys

**DOI:** 10.7759/cureus.83429

**Published:** 2025-05-03

**Authors:** Diane Kuhn, Peter S Pang, Paul I Musey, Chris A Harle

**Affiliations:** 1 Department of Emergency Medicine, Indiana University School of Medicine, Indianapolis, USA; 2 Department of Health Policy and Management, Indiana University Richard M. Fairbanks School of Public Health, Indianapolis, USA

**Keywords:** emergency department, patient-centeredness, patient experience, survey research, value based care

## Abstract

Introduction

Although emergency medicine specialty societies have published policy statements on the use of patient experience data, we know little about how these data are currently used within physician groups. We also have little information about individual emergency physicians’ perspectives on the use of these data.

Methods

A total of 1049 questionnaires were distributed via email to residency program alumni of a large Midwestern residency program in June-July 2024. Participants were asked questions regarding their time in practice since residency, practice setting, and their current group or employer’s use of emergency department (ED) patient experience feedback. Descriptive statistics were calculated for all variables, and a logistic regression was performed to determine associations between respondent sex and years in practice with the odds of perceiving the use of experience ratings as fair. Finally, open-ended responses were reviewed thematically to identify common patterns.

Results

From a distribution to 1049 individuals, there were 99 (9.4%) responses. A total of 33 (33.3%) respondents made free-response comments. The most common uses of data were the publication of group performance and individual emails to physicians regarding patient feedback. Financial incentives were more common at the group level than at the individual level. Female physicians had lower odds of perceiving use of the ratings as fair. In addition, physicians raised concerns in free response comments about gender and racial bias, sample size and distribution of surveys, and the relevance of factors outside of physician control.

Conclusion

Emergency physicians held relatively neutral positions on whether the current use of experience data was reasonable. However, they had numerous concerns regarding the data quality.

## Introduction

Patient experience ratings refer to patient-reported data about their experience of care during a given emergency department (ED) encounter, and these ratings, along with patient comments, are frequently used by physician groups and hospitals for quality improvement. An increasing body of literature shows associations between ED patient experience ratings and factors outside the control of individual physicians and other clinical staff. Specifically, patient demographics and acuity, as well as operational characteristics including wait times, hallway bed use, and National Emergency Department Overcrowding Scale, have all shown associations with ED patient experience [1-3]. While this growing literature represents an important contribution to our understanding of how to measure patient-centered emergency care, it offers limited insight into how ED patient experience ratings and comments are currently used by physician groups and health systems. To address this gap in the literature, we conducted a questionnaire study of emergency physicians to understand how groups and employers utilize ED patient experience ratings. The objective of this study is to understand current uses of patient experience ratings and comments as well as physician perceptions of these data.

## Materials and methods

This was a cross-sectional study conducted in Indiana University, Indianapolis, Indiana, United States. It involved the distribution of a questionnaire to targeted alumni of the emergency medicine residency program. The study was approved by the Indiana University Human Research Protection Program (HRPP) (approval number: 23094). 

Study population

A total of 1,049 copies of the questionnaire were distributed to alumni via email between June and July 2024 using the Qualtrics online platform (Qualtrics International Inc., Seattle, Washington, United States). The alumni contact list included graduates from 1978 to 2023, although the availability of valid email addresses was skewed toward more recent residency graduates. To increase response rates, a single reminder email was sent two weeks after the initial distribution.

Study tool

The questionnaire (see Appendices) was developed by the study team and pilot-tested internally to ensure clarity and face validity [4]. It consisted of both closed- and open-ended questions focused on respondents' perspectives on their primary group’s use of ED patient experience data. In addition, information regarding the number of years in practice, location, and practice settings is provided. Respondents were informed that the estimated time for survey completion was less than five minutes; actual response times were consistent with this estimate. Participation was voluntary and anonymous. Data collection remained open for four weeks. 

Data analysis

Responses were exported from Qualtrics into a comma-separated values (CSV) file that was uploaded into R statistical software version 4.4.3 (R Foundation for Statistical Computing, Vienna, Austria). Descriptive statistics were calculated for all variables, including means and standard deviations for continuous variables and frequencies and percentages for categorical variables. There were 13 missing values for primary practice setting and location, and "Not Provided" was added as a category to account for these missing data. In addition, a binary variable representing a respondent’s belief that use of patient experience ratings was generally fair was developed. The variable was coded 1 if the respondent’s assessment of whether their group’s use of patient experience ratings was fair was either “Definitely yes” or “Probably yes”. The variable was coded zero if the response to fairness was “Might or might not” be fair, “Probably not”, or “Definitely not.” This dichotomization allowed us both to determine the distribution of responses across ordinal categories and to determine associations between predictor variables and an overall perception of fairness. A logistic regression was performed to determine associations between respondent sex and years in practice with the odds of perceiving the use of experience ratings as fair. Finally, open-ended responses were reviewed thematically to identify common patterns. Through an iterative process, these codes were refined and grouped into broader themes based on recurring concepts [5,6].

## Results

Of the 1049 individuals who were sent the questionnaire, there were 99 (9.4%) responses. Table 1 shows descriptive statistics for respondents. The mean years since residency graduation were 13.8 (±8.7).

**Table 1:** Characteristics of Respondents (N=99)

Respondent Characteristic	Distribution, n (%)
Sex	
Female	35 (35.4%)
Male	64 (64.6%)
Primary Practice Location	
Indiana	39 (39.4%)
Iowa	5 (5.1%)
Wisconsin	4 (4.0%)
Colorado	4 (4.0%)
Texas	4 (4.0%)
Other Midwest	10 (10.1%)
West	12 (12.1%)
Southeast	8 (8.1%)
Not Provided	13 (13.1%)
Primary Practice Setting	
Academic	21 (21.2%)
Community Democratic Group	43 (43.4%)
Community Contract Management Group	11 (11.1%)
Hospital Employed	8 (8.1%)
Multiple/Split	3 (3.0%)
Not Provided	13 (13.1%)

Table 2 shows the regression results for perception of fairness as a function of respondent sex and years since graduation. Male respondents had a higher odds of perceiving use of ratings as fair when compared to female respondents, and there was no statistically significant association between years in practice and perception of fairness.

**Table 2:** Logistic Regression Results (N=99)

Respondent Characteristic	Odds Ratio (95% Confidence Interval)
Male Sex	1.33 (1.25, 14.2)
Years in Practice	-0.02 (0.93, 1.04)

**Figure 1:**
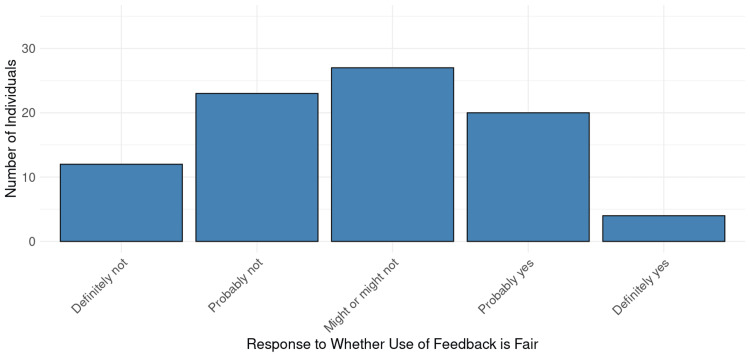
Perspectives on Fairness of Patient Experience Feedback Use

Figure 1 shows the distribution of responses to the question, “Do you feel that your group’s use of patient experience feedback is fair and reasonably represents performance?” Overall, responses were normally distributed with a mode of “Might or might not” be fair.

A total of 33 respondents (33.3%) made free-response comments. Respondents reported a diverse range of uses for patient experience scores and feedback. For individual scores or feedback, 42 (42.4%) respondents reported that emails were sent to individual physicians regarding scores or feedback, 18 (18.2%) reported that individual scores or metrics were published, and seven (7.1%) reported that individual financial incentives were offered. Seventeen individuals (17.2%) reported that individual scores or feedback are not used at all. Twenty-four respondents (24.2%) also reported that group performance had financial incentives, 43 (43.4%) reported that group performance results were shared by email, and 44 (44.4%) stated that group scores or metrics were published. Five respondents (5.1%) reported that group measures were not used. As not all respondents answered each question, and some respondents reported more than one use, overall totals do not represent the total questionnaires completed. Table 3 summarizes free-response comments provided by respondents by sentiment and category. Critical feedback regarding data quality included concerns about sample size and representativeness (surveys only given to discharged patients), as well as perceived gender and racial bias. A separate concern was that the scores reflected operations and processes outside the control of individual emergency physicians.

**Table 3:** Representative Free Response Comments

Topic	Representative Comments
Primarily Negative	
Concerns regarding gender and racial bias	“I am very concerned about gender and racial bias in the patient experience scores, as we know patients display biases in the clinical setting, and this certainly carries over into a subjective evaluation of their “experience” of my (and [the] team’s) performance.” “Patient experience scores are multifactorial, with large swings between shifts. There [are] racial components not factored in, time of day issues, etc. While some extrinsic factors do even out over time, race and gender do not.” “We know there is bias, so utilizing these as incentives seems incorrect.” “P[atien]t experience scores are fraught with bias, racism and faulty data.” “Luckily our scores do not impact much, but they are fully skewed towards the white male MDs in our group.”
Inadequate sample sizes	“Too few patients (5 per provider/month) are sampled for the scores to be of any use.” “...the sample size is not large enough to represent a physician’s overall care.” “Why does a 2-3% response rate dictate policy?” “Response is typically small - <2% of pt volume [and] therefore not meaningful.“
Scores represent information about things outside the control of individual physicians (wait times, boarding, etc.)	“Community shifts are weighted more heavily than academic center shifts because admin[istration] has realized most of the academic center scores are due to environment, boarding, etc.” “Admin[istration] generates the questions and we get dinged even when dissatisfaction is about processes/throughput time. I wish they would get rid of this altogether for emergency medicine.” “I think we all know how heavily flawed these scores are and how minimally they reflect things in the control of the practicing doc[tor].” “Experience scores are completely subjective. A patient could mark a physician down because of factors completely out of their control, i.e. wait times, temperature of the room, etc.”
Surveys only given to discharged patients	“Surveys (Press Ganey at our shop) are only given to discharged patients to my knowledge. Admitted patients or those that elope/[leave] AMA are not surveyed. So the patients who are sick enough to be admitted are omitted from data scores.” “They are only to discharged patients which frequently is not where we spend the bulk of our time and skills.”
Neutral or Positive	
Scores and feedback are used appropriately	“Patient experience scores are tracked by the hospital. If there were any significant outliers, our group would manage that issue.” “General scores and feedback are basically used as an FYI.” “I think the system handles the scores fairly - i.e., we are not directly penalized and I think the system tries to use the scores to identify areas for improvement systemically…”

## Discussion

Overall, our study results suggest that patient experience ratings and feedback are used both at the individual and group level, with emergency physicians’ opinions on whether their use is fair and represents performance approximating a normal distribution centered on the most neutral category. Men were significantly more likely to feel that the use of the ratings was fair compared to women. In addition, respondents expressed concerns in free response comments about potential racial and gender bias. Perceptions of racial and gender bias are most concerning if these perceptions are accurate and scores are used for individual assessment or performance. Racial and gender bias are very challenging to evaluate because they require demonstrating that physicians are evaluated differently by patients based on their personal characteristics in the absence of differences in care provided [7-9]. For example, if patients hold physicians of different races or genders to different standards for what is compassionate, arrogant, or other qualities, then comments across individuals may not be comparable [10]. Some previous literature has found support for this perception through the use of vignettes as well as videotaping of patient interactions, though more work is required [11-13]. While individual performance is not the objective for the Centers for Medicare and Medicaid Services ED patient experience survey, ED Consumer Assessment of Healthcare Providers & Systems (CAHPS), emergency physicians do report that individual scores are used and even occasionally financially incentivized, raising concern that any existing biases in data could unintentionally be translated into disparities in pay or performance assessment.

Beyond concerns about bias, one of the biggest frustrations expressed by emergency physicians is their belief that patient experience ratings reflect factors outside of their, and sometimes even the department’s, control. Physicians perceived operational factors such as wait times, boarding, and throughput as related to experience scores and achievement of incentives but beyond their ability to control, which is well-supported by the literature [1,3,14]. Indeed, literature on the importance of the ED environment and operations in patient experience has given rise to a new area of literature on patient-centered design of EDs to improve privacy and reduce crowding [1,15]. While these efforts are valuable, at present they are in their infancy. As a result, there may be significant differences in the design of EDs and consequent impact on patient experience across facilities. These differences, as well as differences in patient demographics and clinical care such as wait times and use of radiographic studies, limit the extent to which ratings reflect differences in care between clinicians [1-3,14]. Improving patient-centered emergency care and achieving widespread buy-in from emergency physicians will require addressing this issue.

While concerns regarding sample size and distribution to discharged patients may be valid, particularly if scores are used at the individual level or for financial incentives, they may not invalidate the scores as tools for inter-facility comparisons, as low response rates and distribution to discharged patients only are standard across facilities [16]. Continued research on nonresponse, survey mode, and sample representativeness will be important for addressing these issues [17,18].

Limitations of the study

Our study had a number of limitations. First, there was a low response rate (9.4%), and the questionnaire was distributed to the alumni of a single residency program, meaning that the responses may not be representative of all emergency physicians across the country. Furthermore, there may have been self-selection bias in who chose to respond to surveys, which could have skewed our responses to individuals with stronger feelings or more extreme perspectives. In addition, there is not currently a single ED patient experience survey implemented nationally. While most patient experience surveys follow a similar general format, the perspectives presented may be based on slightly different survey items. Finally, our questionnaire, while piloted internally and edited for clarity, is not a previously validated survey instrument. Nonetheless, we feel the importance of understanding physician perspectives on patient experience data outweighs the limitations of our study. This is particularly true given the relatively novel emphasis on these data as a measure of quality and the very limited literature on physician perspectives on their use.

## Conclusions

To the best of our knowledge, this is one of the very few studies describing how ED patient experience surveys are used by physicians. Although emergency physicians held relatively neutral positions overall on whether their group’s use of experience data was reasonable, these perceptions differed across physician genders. In addition, physicians raised numerous concerns in comments, including distribution and sample size, bias, and the relevance of operational variables outside the control of individual physicians. As evidence builds regarding the role of non-clinician factors in ED patient experience, it may become necessary to develop a more comprehensive measure of patient-centered ED care that encompasses elements of care such as wait times, use of hallway beds, ED crowding, and other factors. Measurement of patient-centeredness is now an important aspect of emergency care, and refining these measures will allow us to make more reliable inferences and ultimately care improvements.

## Appendices

**Table 4:** Questionnaire CMG: Crisis Management Group; USACS: US Acute Care Solutions

S. No.	Question	Possible responses
1	How many years have you been in practice since residency graduation?	0-5 years
		6-10 years
		11-15 years
		More than 15 years
2	Which state do you currently practice in primarily?	Indiana
		Other (Please Specify) [free response space provided]
3	How would you describe your current primary practice setting?	Academic
		Community Democratic Group
		Community CMG (TeamHealth, USACS, Envision, etc)
		Other (please specify) [free response space provided]
4	How does your current group use patient experience scores for the group as a whole?	Choose all that apply: Emails are sent to the group as a whole/listserv regarding experience scores and/or patient feedback
		Group scores or metrics are published
		There are group financial incentives (everyone in the group receives the same incentive regardless of whether they individually meet goals)
		Other (please specify) [free response space provided]
5	How does your current group use patient experience scores for individual physicians?	Choose all that apply: Emails are sent to individual physicians regarding personal experience scores and/or patient feedback
		Individual scores or metrics are published
		There are individual financial incentives (individual physicians in the group may receive different financial compensation based on their own performance)
		Other (please specify) [free response space provided]
6	Do you feel that your group’s use of patient experience feedback is fair and reasonably represents performance?	Definitely yes
		Probably yes
		Might or might not
		Probably not
		Definitely not
7	Please provide any additional comments you would like to share regarding how your group uses patient experience scores.	[free response space provided]
